# KAPP—Knowledge, Attitudes, and Practices of Healthcare Professionals on Postpartum Pelvic Floor Dysfunction: A Cross-Sectional Study from Germany

**DOI:** 10.1007/s00192-025-06477-4

**Published:** 2025-12-16

**Authors:** Bettina Blau-Schneider, Esra Bilir, Matthias Kiesel, Anne Scherer-Quenzer, Boris Gabriel, Achim Wöckel, Ulrich Pecks, Kayal Gasimli, Johanna Büchel

**Affiliations:** 1 Department of Obstetrics and Gynecology, University Hospital of Wuerzburg, Josef-Schneider-Strasse 4, 97080 Würzburg, Germany; 2 Department of Obstetrics and Gynecology, St. Josefs-Hospital Wiesbaden, Beethovenstr 20, 65189 Wiesbaden, Germany; 3 Institute of Midwifery, University Hospital of Wuerzburg, Josef-Schneider-Strasse 4, 97080 Würzburg, Germany; 4 Department of Obstetrics and Gynecology, J.W. Goethe University Hospital of Frankfurt, Theodor-Stern-Kai 7, 60596 Frankfurt Am Main, Germany; 5 Department of Gynecology and Obstetrics, University Medical Center Goettingen, Goettingen, Germany; 6 Department of Gynecologic Oncology, Koç University School of Medicine, Istanbul, Türkiye

**Keywords:** Cesarean section, Midwife, Physician, Physiotherapists, Postpartum pelvic floor dysfunction, Survey

## Abstract

**Introduction and Hypothesis:**

Pregnancy and delivery are known risk factors for the development of pelvic floor dysfunction (PFD). An electronic cross-sectional survey was distributed to physicians, midwives, and physiotherapists in Germany, assessing demographics, knowledge, and awareness of postpartum PFD risk/protective factors, and personal or spousal preferences for cesarean section (CS) as a preventive measure. Differences across professional groups were also analyzed.

**Methods:**

An anonymous online survey was conducted via Qualtrics from January 25 to April 15, 2025. The German-language survey targeted healthcare professionals involved in obstetric or postpartum care. Data analysis was performed using SPSS version 28.0 for Mac OS X. Chi-square tests compared binary and categorical variables. *P* values of < 0.05 were considered statistically significant.

**Results:**

After excluding 129 incomplete or non-consented responses, 228 questionnaires were analyzed. The majority of respondents demonstrated a high level of awareness and knowledge regarding the impact of pregnancy and childbirth on pelvic floor health. However, only 36.8% reported routinely providing postpartum counseling for PFD prevention. While 79.8% regularly asked about PFD symptoms postpartum, counseling rates remained low, consistent with earlier findings. Differences emerged between professional groups: physicians were more likely than midwives to view CS as protective against PFD (28.8% vs. 9.3%) and to consider CS for themselves or their partners (27.2% vs. 8.3%). These findings highlight the need to integrate structured PFD counseling protocols into routine antenatal and postnatal care.

**Supplementary Information:**

The online version contains supplementary material available at 10.1007/s00192-025-06477-4

## Introduction

The *International Urogynecological Association (IUGA)* and the *International Continence Society (ICS)* define female pelvic floor dysfunction (PFD) as a clinical condition encompassing symptoms such as urinary incontinence, anorectal dysfunction, pelvic organ prolapse, sexual dysfunction, pelvic pain, and lower urinary tract disorders [1]. PFD represents a significant health issue, affecting nearly 50% of women within a decade of childbirth [2]. In a large cross-sectional study, 43.9% of women reported urinary incontinence, 5.5% experienced pelvic organ prolapse, and 15.6% reported some form of anal incontinence [2]. Another large cross-sectional study found that 46% of women experienced at least one major type of PFD, while nearly 22% reported two or more types [3].

The prevalence of PFD increases with age, ranging from 9.7% in women aged 20–39 to 49.7% in those aged 80 years or older [3]. PFD can significantly impact the quality of life of the affected women [4].

The pathophysiology of PFD is multifactorial. Obesity is a known risk factor, as it exerts chronic pressure on the pelvic floor due to increased intra-abdominal pressure [5, 6]. Additionally, genetic predisposition plays a role, in conjunction with alterations in the collagen structure and composition [7–9].

The effects of pregnancy and childbirth on the development of PFD are well-documented. Pregnancy beyond 20 weeks, regardless of delivery mode, significantly increases the prevalence of major PFD [10]. Most studies have demonstrated a negative impact of vaginal delivery on pelvic floor function [2, 10–12]. While the incidence of urinary incontinence is higher even after uncomplicated vaginal birth, women who undergo elective cesarean section (CS) more frequently report dyspareunia and sexual dysfunction [13].

CS is also an established risk factor for placenta accreta spectrum disorders [14] and for the development of adhesions or cesarean scar niches [15], both of which are associated with increased maternal morbidity and mortality. Therefore, the benefits and risks of each delivery mode must be carefully considered.

It is also noteworthy that nearly 60% of nulliparous women report bothersome symptoms of PFD, suggesting that pregnancy and childbirth are not the only causes [16]. However, vaginal childbirth remains the most significant risk factor for the development of PFD later in life, underscoring the importance of individualized counseling [17].

Spontaneous vaginal birth is significantly more likely in midwife-led care, possibly due to midwives’ emphasis on and expertise in physiologic birth practices [18, 19]. Personalized risk assessment during pregnancy and interdisciplinary collaboration among midwives, physiotherapists, obstetricians, and urogynecologists are essential for preventing PFD and improving quality of care [20].

While extensive literature describes the prevalence and risk factors of PFD, evidence on how healthcare professionals’ knowledge, attitudes, and clinical practices influence postpartum PFD management remains limited. Understanding these professional perspectives is essential to identify gaps in counseling, improve adherence to evidence-based management, and ultimately enhance quality of care.

Therefore, the aim of our study was to assess healthcare professionals’ attitudes, knowledge, and clinical practices regarding postpartum PFD in Germany. Additionally, we examined how these factors differ across professional groups (physicians, midwives, and physiotherapists).

As in recent years, there appears to be growing awareness regarding the impact of pregnancy and childbirth on pelvic floor health, we expected rising counseling rates in comparison with previous studies.

## Materials and Methods

This manuscript was prepared in accordance with the STROBE (Strengthening the Reporting of Observational Studies in Epidemiology) checklist for cross-sectional studies. We conducted a cross-sectional study by means of an anonymous online survey developed and distributed through Qualtrics, a secure, cloud-based survey platform. The survey was conducted in German and targeted healthcare professionals involved in obstetric and/or postpartum care across Germany, including physicians, midwives, and physiotherapists. Data collection was performed between January 25, 2025, and April 15, 2025. Ethical approval for this study was granted by the Institutional Review Board (IRB) of the University Hospital of Wuerzburg (Approval number 2024–280-ka).

The survey started with an electronic informed consent question. We developed a German-language questionnaire comprising 35 multiple-choice items, divided into four sections:

Section 1 (items 1–8): Demographics and professional background, e.g., age, years of experience, and AGUB certification. The German Society of Urogynecology (AGUB e.V.) certifies urogynecologists in Germany at three levels: AGUB I qualifies for conservative, nonsurgical treatment; AGUB II for independently performing complex surgeries, including minimally invasive procedures; and AGUB III for experts with significant clinical and academic contributions. In our study, participants were specifically asked whether they held any of these AGUB certifications.

Section 2 (items 9–20): knowledge on postpartum PFD, Sect. 3 (items 21–26): opinions of predictive tools, and Sect. 4 (items 27–35): counseling practices and considerations for personal or spousal use of CS.

A German online questionnaire regarding knowledge and attitudes related to postpartum PFD was developed for the study, based on a previously published survey by Cooke et al., which had been approved by the executive board of The European Urogynaecology Association (EUGA) [21]. The original English questionnaire was translated into German and reviewed by three midwives, physicians, and physiotherapists, respectively. Following their feedback, the questionnaire was revised accordingly. Subsequently, a back-translation was conducted to verify the consistency and accuracy of the content.

No a priori sample size calculation was performed. As this was an exploratory cross-sectional survey, the sample size was determined by the number of eligible participants who completed the questionnaire during the data collection period.

Data collection was performed using a non-probability sampling approach, combining elements of convenience sampling (open online survey access) and purposive sampling (targeted dissemination via professional associations and networks of midwives, obstetricians, and physiotherapists).

Inclusion criteria were: healthcare professionals involved in antenatal, intrapartum, or postpartum care (midwives, physicians in obstetrics/gynecology, and physiotherapists), currently practicing in Germany, and aged ≥ 18 years.

Exclusion criteria included lack of informed consent, incomplete survey responses (only fully completed data were included), no professional involvement in peripartum care, and duplicate entries (based on timestamp and IP pattern).

All statistical analysis were performed using Statistical Package for Social Sciences (SPSS) version 28.0 for Mac Os X (Chicago, IL, USA). Frequencies and percentiles were used to present descriptive statistics.

To compare binary and categorical variables across groups chi-square test was used. *P* values of < 0.05 were considered statistically significant. No formal correction for multiple testing was applied, as the analyses were exploratory and *p* values are presented descriptively.

ChatGPT version 4.0 (OpenAI Inc., San Francisco, USA) was used for language quality check.

## Results

### Demographics

During the study period, a total of 420 respondents answered the questionnaire. Of these, 129 were excluded due to lack of consent, and 63 incomplete submissions were also removed. As a result, our final analysis included 228 participants.

The demographic characteristics of the survey respondents are summarized in Table 1. The majority of participants (89.9%) were female, 9.2% male, and 0.9% diverse. In terms of professional background, 42.1% were midwives (*n* = 95), 54.9% (*n* = 125) were physicians, and 3.1% (*n* = 7) physiotherapists. There were no responses from student midwives.

**Table 1:** Demographics of the participants

Variable	Number (%)
Gender	
Female	205 (89.9)
Male	21 (9.2)
Other	2 (0.9)
Years of experience	
0–5	47 (20.6)
6–10	48 (21.1)
11–15	44 (19.3)
16–20	29 (12.7)
21–25	18 (7.9)
> 25	42 (18.4)
Role	
Resident	30 (13.2)
Attending	44 (19.3)
Senior attending	46 (20.2)
Chief	5 (2.2)
Midwife student	0 (0.0)
Midwife	96 (42.1)
Physiotherapist	7 (3.1)
Certification*	
AGUB I	8 (8.4)
AGUB II	4 (4.2)
AGUB III	4 (4.2)
Subspeciality in perinatology (*n* = FA + OA + CA)	
Yes	28 (29.5)
No	67 (70.1)
Clinical setting	
State hospital	31 (13.6)
Academic teaching hospital	53 (23.2)
University Hospital	105 (46.1)
Outpatient clinic	60 (26.3)
Other	17 (7.5)
Clinical activity †	
Prenatal care	176 (77.2)
Postnatal care	176 (77.2)
Care/management of births	165 (72.4)
Conservative urogynaecological therapy	77 (33.8)
Surgical urogynaecological therapy	41 (18.0)
Certification as pelvic floor center	
Yes	106 (46.5)
No	88 (38.6)
Not applicable	34 (14.9)

^*^ German Society of Urogynaecology (AGUB e.V.)

^†^ multiple selections were allowed

Among the physicians, only 8% of the respondents were AGUB-certified urogynecologists with 8.4%, 4.2%, and 4.2% for AGUB 1, 2, and 3, respectively.

Regarding workplace settings, 46.1% of participants were employed at university hospitals, 23.2% at academic teaching hospitals, 13.6% at general hospitals, and 26.3% in outpatient practices. Participants’ professional responsibilities related to postpartum PFD included prenatal and postnatal care (77.2%), birth management (72.4%), and conservative urogynecological therapy (33.8%). Furthermore, 46.5% of respondents reported working in certified pelvic floor centers.

### Knowledge About Risk Factors, Prevention, and Management of Postpartum PFD

**Fig. 1:**
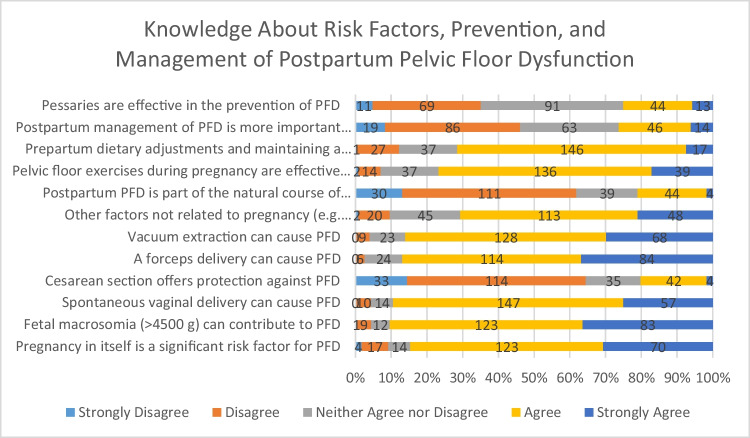
Knowledge about risk factors, prevention, and management of postpartum PFD (the numbers on the bars represent the total numbers for the corresponding scale) *BMI* Body mass index

The knowledge about risk factors, prevention, and management of postpartum PFD are presented in Fig. 1.

A vast majority of participants (84.6%, *n* = 193) (strongly) agreed that pregnancy itself constitutes a significant risk factor for PFD. Similarly, 89.4% (*n* = 206) identified fetal macrosomia (birth weight > 4500 g) as a contributing factor. In addition, 89.5% (*n* = 204) agreed that spontaneous vaginal delivery increases the risk of PFD later in life. Noteworthily, 19.7% (*n* = 46) believed that cesarean section has a protective effect, while 64.5% (*n* = 147) (strongly) disagreed with this assumption.

Interestingly, the impact of instrumental deliveries on PFD was not perceived as being more deleterious than that of spontaneous vaginal birth: 86.8% (*n* = 198) and 85.9% (*n* = 196) of respondents estimated that forceps and vacuum extraction, respectively, were associated with a comparable risk profile for the pelvic floor such as vaginal birth.

Forty-eight (21.1%) of respondents assumed that postpartum PFD represents a natural consequence of pregnancy and is not preventable regardless of delivery mode, while 61.9% (*n* = 141) (strongly) disagreed with this view. Likewise, only 26.3% (*n* = 60) considered the postpartum management of PFD to be more important than prevention.

The effectiveness of pelvic floor muscle exercises during pregnancy for the prevention of PFD was affirmed by 76.7% (*n* = 175) of participants. Similarly, 71.5% (*n* = 163) endorsed prepartum dietary measures and maintaining a normal body mass index as preventive measures. Of note, the benefit of using pessaries for the prevention of PFD was estimated differently by the respondents: 35.1% (*n* = 80) (strongly) disagreed with their efficacy, whereas 24.9% (*n* = 57) (strongly) supported their use.

### Attitudes Toward the Prediction and Clinical Relevance of Postpartum PFD

**Fig. 2:**
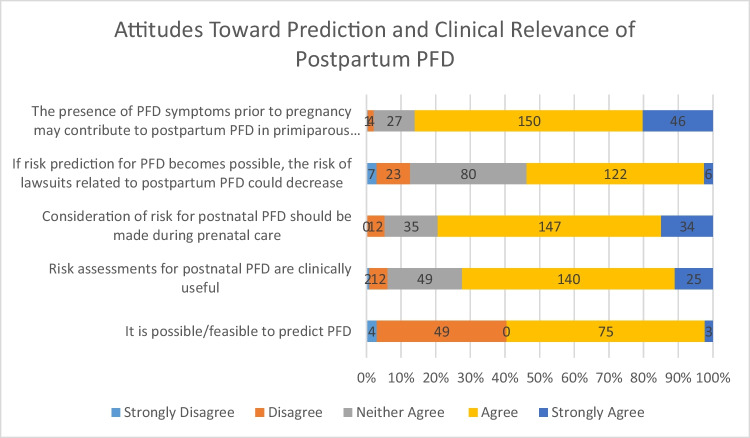
Attitudes toward prediction and clinical relevance of postpartum PFD (the numbers on the bars represent the total numbers for the corresponding scale)

Figure 2 outlines attitudes toward the prediction and clinical relevance of postpartum PFD.

A considerable number of participants (42.5%, *n* = 97) expressed uncertainty about the feasibility of predicting PFD; 34.2% (*n* = 78) believed prediction is possible, while 23.3% (*n* = 53) disagreed.

Regarding the clinical usefulness of a risk assessment, 72.4% (*n* = 165) affirmed its value, whereas 6.2% (*n* = 14) (strongly) disagreed. A majority (79.4%, *n* = 181) supported the inclusion of a PFD risk estimation into the prenatal care, which was supported by 14.9% (*n* = 34) of respondents strongly agreeing to this statement. More than half of the participants (51.7%, *n* = 118) believed that improved prediction could reduce litigation issues related to postpartum PFD, whereas only 13.2% (*n* = 30) strongly disagreed. When asked whether high-risk patients should be offered CS as a preventive measure, 45.6% (*n* = 104) (strongly) agreed and 26.3% (*n* = 60) disagreed. Most participants (86%, *n* = 196) agreed that pre-existing PFD symptoms may increase the postpartum risk of PFD in primiparous women.

A total of 43.4% (*n* = 99) reported routinely inquiring about PFD symptoms during pregnancy, while 56.6% (*n* = 129) did not. Recommendations for prenatal pelvic floor exercises were made by 48.2% (*n* = 110), with 51.8% (*n* = 118) not providing such advice. PFD prevention influenced delivery-related decision-making in 36.8% (*n* = 84) of participants, while 63.2% (*n* = 144) reported it did not. CS without obstetric indication to prevent PFD was considered by 51.3% (*n* = 117), whereas 48.7% (*n* = 111) rejected this approach. Postpartum counseling for PFD prevention was routinely offered by 36.8% (*n* = 84), and not provided by 63.2% (*n* = 144). A majority (79.8%, *n* = 182) asked patients about postpartum PFD symptoms, while 20.2% (*n* = 46) did not. Postpartum pelvic floor exercises were recommended by 95.2% (*n* = 217).

Pessaries were recommended for “at-risk” postpartum patients by 34.2% (*n* = 78), while 65.8% (*n* = 150) did not recommend their use. Only 19.3% (*n* = 44) would consider a cesarean section for themselves or their partner to prevent future PFD; 80.7% (*n* = 184) would not.

Knowledge regarding risk factors for PFD did not differ significantly between the professional groups. Differences between physicians and midwives were observed regarding the perceived impact of pregnancy and childbirth on the risk of later PFD. Physicians were more likely to attribute a protective effect to CS, with a rate of 28.8% agreeing or strongly agreeing, compared to only 9.3% of midwives. Furthermore, 27.2% of physicians stated they would consider a CS to protect the pelvic floor for themselves or their partner, whereas only 8.3% of midwives would do so. These differences were significant (Table 2).

**Table 2:** (Strong) Agreement for the statement in physicians and midwifes, p-value chi-square test

	Physicians (*n* = 125)	Midwifes (*n* = 96)	*p* value chi-square test
Pregnancy in itself is a significant risk factor for PFD	115 (92.0%)	72 (75.0%)	0.022
Fetal macrosomia (> 4500 g) can contribute to PFD	121 (96.8%)	78 (81.3%)	0.190
Spontaneous vaginal delivery can cause PFD	121 (96.8%)	76 (79.2%)	0.001
Cesarean section offers protection against PFD	36 (28.8%)	9 (9.3%)	< 0.001
A forceps delivery can cause PFD	118 (94.4%)	74 (77.1%)	< 0.001
Vacuum extraction can cause PFD	113 (90.4%)	78 (81.3%)	0.357
Other factors not related to pregnancy (e.g., smoking, BMI) are more important causes of PFD	97 (77.6%)	58 (60.4%)	0.188
Postpartum PFD is part of the natural course of pregnancy and is not preventable, regardless of the mode of delivery	37 (15.2%)	44 (45.9%)	0.021
Pelvic floor exercises during pregnancy are effective in preventing PFD	92 (73.6%)	77 (80.2%)	0.361
Prepartum dietary adjustments and maintaining a normal BMI may prevent PFD	91 (72.8%)	69 (71.9%)	0.464
Postpartum management of PFD is more important than prevention	27 (21.6%)	31 (32.3%)	0.073
Pessaries are effective in the prevention of PFD	33 (26.4%)	21 (21.9%)	0.239
It is possible/feasible to predict PFD	50 (40.0%)	24 (25.0%)	0.037
Risk assessments for postnatal PFD are clinically useful	105 (84.0%)	56 (58.3%)	0.014
Consideration of risk for postnatal PFD should be made during prenatal care	109 (87.2%)	66 (68.8%)	0.677
If risk prediction for PFD becomes possible, the risk of lawsuits related to postpartum PFD could decrease	73 (58.4%)	39 (40.6%)	0.128
If high-risk patients can be reliably identified, it makes sense to offer a cesarean section	70 (56.0%)	64 (66.7%)	0.289
The presence of PFD symptoms prior to pregnancy may contribute to postpartum PFD in primiparous women	117 (93.6%)	76 (79.2%)	0.015
I routinely ask about PFD symptoms in pregnancy	48 (38.4%)	46 (47.9%)	0.156
I routinely recommend pelvic floor exercises before birth to prevent PFD	52 (41.6%)	51 (53.1%)	0.089
Prevention of PFD influences my decisions during childbirth	41 (32.8%)	40 (41.7%)	0.175
I would offer a cesarean section in selected cases to prevent PFD without obstetric indication	81 (64.8%)	32 (33.3%)	< 0.001
I routinely offer counseling to prevent postpartum PFD	49 (39.2%)	30 (31.3%)	0.222
I routinely ask about postpartum PFD symptoms	93 (74.4%)	82 (85.4%)	0.046
I routinely recommend pelvic floor exercises after birth	115 (92.0%)	95 (99.0%)	0.018
I advise at-risk patients to use pessaries postpartum	49 (39.2%)	26 (26.4%)	0.040
I would consider a cesarean section for myself or my partner to avoid future PFD	34 (27.2%)	8 (8.3%)	< 0.001

*BMI* body mass index, *PFD* Pelvic floor dysfunction

Physiotherapists were excluded due to the small sample size (*n* = 7)

The usefulness of pessaries was estimated consistently as being effective by 26.4% of physicians and 21.9% of midwives. Nevertheless, pessary use was less frequently recommended by midwives compared to physicians (26.4% vs. 39.2%, respectively, *p* = 0.04).

## Discussion

Our study provides a comprehensive assessment of healthcare professionals’ knowledge, attitudes, and practices regarding postpartum PFD in Germany, integrating perspectives from physicians, midwives, and physiotherapists.

### Knowledge

The majority of participants demonstrated a high level of awareness regarding the impact of pregnancy and childbirth on pelvic floor health, indicating a comparatively strong knowledge base.

This contrasts with earlier surveys of less specialized healthcare professionals: Huang et al. found that only about half of general gynecologists and obstetricians had adequate knowledge of PFD, though many expressed interest in further education [22].

Similarly, general practitioners reported low confidence in diagnosing incontinence causes [23], and Mazloomdoost et al. found that primary care providers demonstrated good awareness and management of urinary incontinence (UI) and overactive bladder (OAB), but frequently underestimated pelvic organ prolapse (POP) and rarely screened for it [24].

Our findings align with previous studies showing that obstetrics and urogynecology professionals generally possess good knowledge and awareness on PFD, athough this does not always translate into clinical routine [21, 25].

These results suggest that while knowledge and awareness are strong, especially among specialized professionals, translation into routine practice may remain limited.

### Attitudes

Participants generally held positive attitudes toward risk assessment tools and preventive strategies. For example, 61.4% valued the UR-CHOICE risk calculator [17] for individualized counseling, and 64.5% supported including PFD risk assessment in prenatal care, consistent with findings from Cooke et al. and Chen et al. [21, 25]. Despite this, attitudes toward cesarean section as a preventive strategy varied: only 20.2% considered CS protective against PFD, though over half viewed elective CS for high-risk patients as acceptable.

### Practices (Counseling and Interventions)

#### PFD Counselling

Despite high knowledge and positive attitudes, counseling practices were limited. Fewer than half of respondents (43.4%, *n* = 99) reported routinely inquiring about PFD symptoms during pregnancy, with higher rates among midwives than physicians (47.9% vs. 38.4%). Postpartum assessment was more common, reported by 85.4% of midwives and 74.4% of physicians. However, counseling on PFD-related issues was routinely provided by only a minority of respondents, with 39.2% of physicians and 31.3% of midwives.

Despite well-established evidence supporting pelvic floor muscle training (PFMT) during pregnancy and postpartum [26], only 41.6% of physicians and 53% of midwives recommended antenatal PFMT. In contrast, postpartum PFMT was widely endorsed (92% of physicians, 99% of midwives).

This gap mirrors prior findings: Cooke et al. reported that despite high knowledge, one-third of urogynecologists did not enquire about PFD symptoms during pregnancy, 25% failed to do so postpartum, and 39% did not routinely provide counseling on prevention [21].

Comparisons with studies on obstetricians showed similar patterns. In a cohort of Chinese obstetricians nearly 30% never inquired about symptoms, and approximately 50% rarely or never provided guidance on PFMT [25].

A US survey by Dessie et al. found that over half of 173 obstetric providers never addressed postpartum urinary incontinence, and 73.7% never discussed fecal incontinence. Residents were less likely to counsel than attendings, with key barriers being time constraints (39.9%) and lack of information (30.1%) [27].

As current counseling practices show little improvement over previous data, new strategies are needed.

It may be necessary to integrate counseling on PFD into routine prenatal care, as recommended by the German Maternity Guidelines.

Offering appropriate reimbursement could enhance preventive care and reduce long-term healthcare costs.

#### Pessary Use

Although the use of pessaries in the postpartum period is feasible and well-accepted, especially in woman with impaired pelvic floor function [28], a randomized controlled trial for its effectiveness is still lacking. This explains the low recommendation rate, with midwives recommending the use less often than physicians (26.4% vs. 39.2%, respectively, *p* = 0.040).

#### Risk Estimate Tools

The online UR-CHOICE risk calculator (www.riskcalc.org) is a validated tool for predicting a woman’s future risk of pelvic floor dysfunction (PFD). It supports individualized counseling by stratifying women into risk categories and comparing outcomes of vaginal delivery versus cesarean section. The tool incorporates key risk factors such as pre-pregnancy incontinence, maternal age, BMI, family history, birth weight, and maternal height [17].

Regarding the clinical utility of risk assessment, 61.4% (*n* = 140) affirmed its value, while only 0.9% (*n* = 2) strongly disagreed. A majority (64.5%, *n* = 147) supported the inclusion of PFD risk consideration in prenatal care, with 14.9% (*n* = 34) strongly agreeing.

This is in line with previous studies where most of the respondents had positive attitudes regarding risk-estimate tools for PFD risk. In the study of Cooke et al. tools for estimating the risk of PFD were viewed positively by nearly 60% of participants and could help standardize and improve counseling [21]. Similarly, in the study by Chen et al., the majority of respondents expressed positive attitudes toward the use of risk assessment tools for PFD as well as toward prevention strategies [25].

#### Influence on Litigation

Given the importance of ensuring that women are well-informed about the risks and benefits of treatment options, it appears plausible that a risk prediction tool could help in reducing legal actions against healthcare providers involved in postpartum PFD counseling in the future.

Almost half of the respondents in our study (49.1%, *n* = 112) assumed that improved risk prediction could contribute to a reduction in litigation related to postpartum PFD, while only 3.1% (*n* = 7) strongly disagreed. This proportion is notably higher than in the study by Cooke et al., in which only 30% of participants agreed that risk prediction tools could help mitigate litigation related to postpartum PFD [21]. It can be hypothesized that clinicians assume pregnant women are increasingly well-informed about the relationship between childbirth and pelvic floor health.

#### Role of Cesarean Section

In the present study, only 20.2% (*n* = 46) of respondents considered CS to be protective against PFD, whereas 64.5% (*n* = 147) disagreed with this assumption. Despite this, more than half of the participants (51.3%) deemed a CS without obstetric indication to be an appropriate option for the prevention of PFD in high-risk patients, while 48.7% (*n* = 111) did not support this approach.

Notably, only 19.3% (*n* = 44) reported that they would personally consider a CS for themselves or their partner with the specific aim of preventing future PFD. This perspective was more frequently expressed by physicians, of whom 27.2% indicated such a consideration, compared to only 3.0% of midwives, highlighting a substantial difference in attitudes between professional groups (*p* < 0.001). The difference between male and female physicians regarding the preference for elective CS was not significant (24.3% vs. 38.1% respectively, *p* = 0.191).

In a postal survey of 282 obstetricians in Great Britain, 17% indicated they would choose an elective CS without medical indication, mainly due to concerns about perineal trauma. Female obstetricians were more likely than male obstetricians to prefer CS (31% vs. 8%). However, when faced with a scenario involving a mid-cavity instrumental delivery, only 5% favored CS [29]. Similarly, in the study by Cooke et al., CS was the least accepted preventive strategy among the surveyed urogynecology team; just over 50% of respondents considered CS to be a protective factor against PFD. Of the respondents, 35% were willing to offer a CS in selected cases for the prevention of some form of postnatal PFD, even in the absence of obstetric complications, and a quite low number of nearly 25% would prefer CS for themselves or for their spouse for postnatal PFD prevention [21].

In a survey among German obstetricians and gynecologists from 2005, only 10% of the participants chose vaginal delivery for themselves or their partner as best medical practice [30]. According to our data, this rate has nearly tripled over the past 30 years in Germany and appears to vary across countries. Nevertheless, the majority of participants expressed a preference for vaginal delivery. This decision is likely influenced not only by immediate perioperative risks, such as hemorrhage and injury to adjacent organs, but also by potential long-term implications for subsequent pregnancies, including placenta accreta spectrum, uterine rupture, and reduced fertility.

### Strenght and Limitations

While the findings provide valuable insights into this important topic, certain limitations must be acknowledged. A limitation of this study is the absence of an a priori sample size calculation, which may limit the statistical power to detect smaller between-group differences. As the survey was distributed exclusively online and limited to one country, potentially affecting the representativeness and generalizability of the results. Furthermore, owing to the dissemination strategy—via email, newsletters, and online platforms—the total number of healthcare providers who had theoretical access to the questionnaire remains unknown, rendering the assessment of a response rate impossible. Consequently nonresponse bias cannot be excluded. Social desirability bias may also have influenced self-reported counseling practices, potentially leading to overestimating counselling rates. In addition, the online distribution may have introduced selection bias, as healthcare professionals who are more engaged, interested, or knowledgeable about postpartum PFD may have been more likely to respond. This could result in an overestimation of awareness, attitudes, and counseling practices, limiting the generalizability of our findings. Future studies should consider alternative recruitment strategies to obtain a more representative sample and better assess generalizability. A further limitation is the limited number of physiotherapists included in the study (*n* = 7), which precluded a separate analysis of the responses from this subgroup.

Multiple chi-square tests were performed without formal correction for multiple testing, which may increase the risk of type I error.

## Conclusion

This study represents a unique investigation with a comparatively large sample size, examining the knowledge, attitudes, and counseling practices of physicians, midwives, and physiotherapists regarding postpartum PFD. The multiprofessional perspective offers a more comprehensive view of preventive approaches.

Consistent with previous studies, the majority of participants demonstrated a high level of awareness and knowledge about the impact of pregnancy and childbirth on pelvic floor health, with no significant differences between physicians and midwives. Despite this, counseling rates on postpartum PFD remained relatively low consistent with previous studies.

While our study did not directly assess reasons for this gap, prior research highlights time constraints as a major barrier [27].

Further studies are warranted to determine whether this constitutes a key factor contributing to the insufficient counseling on PFD among healthcare providers in Germany.

These findings highlight the need to integrate structured PFD counseling protocols into routine antenatal and postnatal care, in line with the German Maternity Guidelines, alongside strengthened interdisciplinary collaboration. Implementation could be further supported through appropriate reimbursement models to incentivize counseling. This approach creates a coherent narrative linking our survey findings to actionable strategies for improving postpartum PFD management. Early and comprehensive counseling has the potential not only to enhance maternal health outcomes but also to reduce the long-term healthcare burden associated with PFD.

## Supplementary Information

Below is the link to the electronic supplementary material.

Supplementary file1 (DOCX 22 KB)

## Data Availability

The data underlying this article cannot be shared publicly due to patient privacy regulations. Data are available from the corresponding author upon reasonable request.
