# Supplementary material for: KAPP—Knowledge, Attitudes, and Practices of Healthcare Professionals on Postpartum Pelvic Floor Dysfunction: A Cross-Sectional Study from Germany
Source: Int Urogynecol J. 2025 Dec 16;37(5):1357–66. doi: 10.1007/s00192-025-06477-4 (PMC13226384; doi:10.1007/s00192-025-06477-4)
Supplement: Supplementary file 1 — Supplementary file1 (DOCX 22 KB) [file 192_2025_6477_MOESM1_ESM.docx]

Supplementary: **The German survey questions**

**DEMOGRAFIE**

1. Wie lange praktizieren Sie bereits (in Jahren)?
a. 0-5
b. 6-10
c. 11-15
d. 16-20
e. 21-25
f. >25

2.Arbeiten Sie als ?
a. WeiterbildungsassistentIn Gynäkologie/Geburtshilfe
b. Facharzt/-ärztin Gynäkologie/Geburtshilfe
c. Oberarzt/ärztin Gynäkologie/Geburtshilfe

d. Chefarzt/ärztin Gynäkologie/Geburtshilfe

e. Hebammenstudierende

f. Hebamme

g. PhysiotherpeutIn

3.Haben Sie eine Zertifizierung für die Urogynäkologie
a. nein
b. AGUB I
c. AGUB II
d. AGUB III

4. Haben Sie die Schwerpunktbezeichnung Spezielle Geburtshilfe und Perinatalmedizin

a. ja

b. nein

5.Geschlecht
a. Männlich
b. Weiblich

c. Divers

6. Klinisches Umfeld
a. Allgemeine Klinik
b. Akademisches Lehrkrankenhaus
c. Uniklinik
d. Praxis

e. Sonstiges

7.Umfasst Ihre Tätigkeit (Mehrfachnennung möglich):
a. Pränatale Versorgung
b. Postnatale Versorgung

c. Betreuung/ Leitung von Geburten

d. konservative urogynäkologische Therapie

e. operative urogynäkologische Therapie

7. Gibt es in Ihrer Klinik ein zertifiziertes Beckenbodenzentrum

a. ja

b. nein

c. nicht zutreffend

**B: Einschätzung des Einfluss von Schwangerschaft und Geburt auf Beckenbodenbeschwerden**

Bitte geben Sie auf der folgenden Skala an, wie sehr Sie jedem der Aussagen zustimmen: Stimme überhaupt nicht zu Stimme nicht zu Weder noch Stimme zu Stimme vollkommen zu

Schwangerschaft an sich ist ein signifikanter Risikofaktor für PFD.

Eine fetale Makrosomie (>4500 g) kann zu PFD beitragen.

Eine spontane vaginale Geburt kann PFD verursachen.

Ein Kaiserschnitt bietet Schutz vor PFD.

Eine Zangengeburt kann PFD verursachen.

Eine Saugglockengeburt kann PFD verursachen.

Andere Faktoren, die nicht mit der Schwangerschaft zusammenhängen (z. B. Rauchen, Body-Mass-Index), sind wichtigere Ursachen für PFD.

Postpartale PFD ist Teil des natürlichen Verlaufs der Schwangerschaft und nicht vermeidbar, unabhängig von der Art der Entbindung.

Beckenbodenübungen in der Schwangerschaft sind wirksam bei der Prävention von PFD.

Eine präpartale Anpassung der Ernährung und die Aufrechterhaltung eines normalen BMI können möglicherweise PFD verhindern.

Das postpartale Management von PFD ist wichtiger als die Prävention.

Pessare sind wirksam in der Prävention von PFD.

**C: ASPEKTE DER KLINISCHEN PRAXIS ZUR VORHERSAGE VON PFD UND DER UMSETZBARKEIT DER PRÄNATALEN RISIKOVORHERSAGE**

Bitte geben Sie auf der folgenden Skala an, wie sehr Sie jedem der Aussagen zustimmen: Stimme überhaupt nicht zu Stimme nicht zu Weder noch Stimme zu Stimme vollkommen zu

Es ist möglich/realisierbar, PFD vorherzusagen.

Risikoabschätzungen für postnatale PFD sind klinisch nützlich.

Eine Berücksichtigung des Risikos für postnatale PFD sollte bei der pränatalen Versorgung erfolgen.

Wenn eine Risikovorsage für PFD möglich wird, könnte das Risiko von Klagen im Zusammenhang mit postpartaler PFD sinken.

Wenn Hochrisikopatient:innen zuverlässig identifiziert werden können, ist es sinnvoll, einen Kaiserschnitt anzubieten.

Das Vorliegen von PFD-Symptomen vor der Schwangerschaft kann bei primiparen Frauen zu postpartaler PFD beitragen. **D: klinisches Management**

Ich frage routinemäßig nach PFD-Symptomen in der Schwangerschaft.
a. Ja
b. Nein

Ich empfehle routinemäßig vor der Geburt Beckenbodenübungen zur Prävention von PFD.
a. Ja
b. Nein

Die Prävention von PFD beeinflusst meine Entscheidungen unter der Geburt.

a. Ja

b. Nein

Ich würde in ausgewählten Fällen einen Kaiserschnitt zur Prävention von PFD ohne geburtshilfliche Indikation anbieten.
a. Ja
b. Nein

Ich biete routinemäßig Beratung zur Prävention von postpartaler PFD an.
a. Ja
b. Nein

Ich frage routinemäßig nach PFD-Symptomen nach der Geburt.
a. Ja
b. Nein

Ich empfehle routinemäßig nach der Geburt Beckenbodenübungen.
a. Ja
b. Nein

Ich rate Risikopatientinnen zur Anwendung von Pessaren postpartal

a: Ja

b. Nein

Ich würde für mich selbst oder meine Partnerin einen Kaiserschnitt in Betracht ziehen, um künftige PFD zu vermeiden.
a. Ja
b. Nein
